# Low-Dose Metronomic Chemotherapy With Fixed Low-Dose Nivolumab for Recurrent Cervical Cancer After Radiotherapy: Experience From Routine Clinical Practice

**DOI:** 10.7759/cureus.105439

**Published:** 2026-03-18

**Authors:** Goutham Reddy Ramidi, Rajan Yadav, Goutham Sunny, Abinash Patnaik

**Affiliations:** 1 Medical Oncology, RVM Institute of Medical Sciences, Siddipet, IND; 2 Medical Oncology, Gujarat Cancer and Research Institute, B. J. Medical College, Ahmedabad, IND

**Keywords:** chemo-immunotherapy treatment, low dose immunotherapy, metronomic chemotherapy, nivolumab, recurrent carcinoma cervix

## Abstract

Background

Recurrent cervical cancer following definitive radiotherapy remains a difficult clinical problem, particularly in low- and middle-income countries. Although immune checkpoint inhibitors have shown activity in this setting, their cost and prolonged treatment duration limit real-world accessibility. Strategies that retain biological activity while improving affordability are therefore needed.

Methods

We conducted a retrospective review of 21 patients with recurrent cervical cancer previously treated with radiotherapy or chemoradiotherapy. Patients received a fixed low dose of nivolumab (20 mg intravenously every two weeks) combined with metronomic oral chemotherapy using either methotrexate or capecitabine. Clinical outcomes included progression-free survival, overall survival, response rates, and toxicity.

Results

The overall response rate was 38%, seen in eight patients, and disease control was achieved in 14 (67%). Median progression-free survival was approximately 8.6 months, and median overall survival was about 15 months. One-year and two-year overall survival rates were 61% and 42%, respectively. Treatment was generally well tolerated, with mostly grade 1/2 adverse events and no treatment-related deaths.

Conclusion

Low-dose nivolumab combined with metronomic chemotherapy appears to be a reasonable and pragmatic option for selected patients with recurrent cervical cancer, particularly in settings where access to standard immunotherapy is limited. These observations support further prospective evaluation.

## Introduction

Cervical cancer continues to represent a major global health burden, with a disproportionate impact on women in low- and middle-income countries (LMICs). Despite advances in screening and vaccination, many patients still present with locally advanced disease. Definitive radiotherapy, often combined with concurrent platinum-based chemotherapy, remains the cornerstone of treatment. While initial disease control is frequently achieved, recurrence after radiotherapy remains a common concern and is associated with poor outcomes [1].

Management of recurrent cervical cancer is challenging. Most patients have already been exposed to platinum-based chemotherapy, limiting subsequent treatment options. Conventional cytotoxic regimens provide only modest benefit, with short progression-free intervals and cumulative toxicity. The addition of bevacizumab to chemotherapy has improved survival in selected patients, but its toxicity profile and limited availability restrict widespread use in routine practice [2].

Immune checkpoint inhibitors targeting the programmed death-1 (PD-1) pathway have emerged as an important therapeutic option in recurrent and metastatic cervical cancer. Trials evaluating nivolumab and pembrolizumab have demonstrated durable responses in a subset of patients, particularly those with programmed death-ligand 1 (PD-L1)-positive tumors [3,4]. However, overall response rates remain modest, and full-dose immunotherapy is associated with substantial financial burden, making sustained treatment difficult in LMIC settings.

An important observation from early pharmacokinetic and pharmacodynamic studies is that PD-1 receptor occupancy occurs at doses far lower than those commonly used in clinical trials. Near-maximal receptor saturation has been demonstrated at low nivolumab doses, with higher doses offering little additional biological benefit [5]. This raises a clinically relevant question: whether fixed low-dose regimens could retain immunologic activity while improving affordability and access.

Parallel to this, there has been renewed interest in metronomic chemotherapy, defined as the continuous administration of low-dose oral chemotherapy. Beyond direct cytotoxic effects, metronomic regimens exert anti-angiogenic effects and modulate the tumor immune microenvironment [6]. Low-dose methotrexate has been shown to reduce regulatory T-cell activity, while metronomic capecitabine selectively depletes myeloid-derived suppressor cells, thereby favoring antitumor immune responses [7,8].

In everyday oncology practice, particularly outside clinical trials, treatment decisions are often shaped as much by feasibility and cost as by efficacy. It was in this context that the present treatment approach evolved. In this study, we report our real-world experience with fixed low-dose nivolumab combined with metronomic methotrexate or capecitabine in patients with recurrent cervical cancer following radiotherapy.

## Materials and methods

Study design

This was a retrospective observational study conducted in routine clinical practice. Medical records of patients treated between January 2022 and December 2024 were reviewed. The study reflects real-world decision-making rather than a predefined protocol-driven approach.

Patient selection

Eligible patients were adult women with histologically confirmed recurrent cervical cancer after prior radiotherapy or concurrent chemoradiotherapy. All patients had an Eastern Cooperative Oncology Group (ECOG) performance status of 0-2 and adequate organ function. Patients with prior exposure to immune checkpoint inhibitors were excluded. PD-L1 testing was not routinely available and was not mandatory for treatment initiation.

Treatment protocol

All patients received nivolumab at a fixed dose of 20 mg administered intravenously every two weeks. In addition, patients received one of the following metronomic chemotherapy regimens: methotrexate 10 mg orally twice weekly or capecitabine 500 mg orally twice daily.

The choice of metronomic agent was influenced by physician preference, prior tolerance, comorbidities, and patient convenience. Treatment was generally continued until radiological progression or clear clinical decline, although in a small number of cases, decisions were individualized based on patient preference and overall condition.

Outcome measures

The primary endpoint was progression-free survival (PFS), defined as the time from treatment initiation to disease progression or death. Secondary endpoints included overall survival (OS), objective response rate (ORR), disease control rate (DCR), and treatment-related toxicity.

Tumor responses were assessed using routine imaging and standard response criteria. Adverse events were graded according to the Common Terminology Criteria for Adverse Events.

Statistical analysis

Descriptive statistics were used to summarize baseline characteristics and outcomes. Survival outcomes were estimated using the Kaplan-Meier method. Given the exploratory nature of the study, no formal hypothesis testing was planned.

## Results

Patient characteristics

A total of 21 patients were included, and their baseline characteristics are summarized in Table 1. The median age was 52 years (range = 38-68). A total of 17 (81%) patients had squamous cell carcinoma. All had received prior platinum-based chemotherapy as part of definitive treatment.

**Table 1:** Baseline patient characteristics.

Characteristic	Number (%)
Median age (range)	52 (38-68)
Squamous histology	17 (81)
Local recurrence only	9 (43)
Nodal recurrence	7 (33)
Distant metastases	5 (24)
Prior platinum therapy	21 (100)

Treatment exposure

The median duration of treatment was 6.5 months. Patients received a median of 10 doses of nivolumab. Compliance with oral metronomic chemotherapy was generally good.

Tumor response

Best tumor responses are summarized in Table 2. Two (10%) patients achieved a complete response, and six (28%) achieved a partial response, resulting in an overall response rate of 38%. Six (29%) additional patients had stable disease, yielding a disease control rate of 67%. Seven (33%) patients experienced progressive disease as the best response.

**Table 2:** Best tumor response.

Response	Overall, n (%)	Methotrexate (n = 11)	Capecitabine (n = 10)
Complete response	2 (10)	1	1
Partial response	6 (28)	3	3
Stable disease	6 (29)	4	2
Progressive disease	7 (33)	3	4
Objective response rate	38%	36%	40%
Disease control rate	67%	64%	70%

Response patterns appeared broadly similar between the two metronomic backbones, although the small sample size precludes meaningful comparison.

Survival outcomes

Survival outcomes are summarized in Table 3. Median PFS was approximately 8.6 months (95% CI: 6.2-11.0). Median overall survival was about 15.2 months (95% CI: 11.8-18.6). One-year overall survival was 61%, and two-year overall survival was 42%.

**Table 3:** Survival outcomes.

Outcome	Value
Median progression-free survival	8.6 months
Median overall survival	15.2 months
1-year overall survival	61%
2-year overall survival	42%

Overall survival according to the site of recurrence is summarized in Table 4, which shows that patients with isolated local recurrence experienced longer survival compared with those with nodal or distant disease.

**Table 4:** Overall survival by site of recurrence.

Site of recurrence	Median overall survival (months)
Local	18.4
Nodal	15.1
Distant	11.6

Safety and tolerability

Treatment-related adverse events are summarized in Table 5. Treatment was generally well tolerated. Most adverse events were grade 1-2. Fatigue, mucositis, diarrhea, hand-foot syndrome, and hypothyroidism were the most commonly observed toxicities. One (5%) patient experienced grade 3 hand-foot syndrome. No treatment-related deaths occurred.

**Table 5:** Treatment-related adverse events.

Adverse event	Grade 1-2 (%)	Grade ≥3 (%)
Fatigue	6 (29)	1 (5)
Mucositis	4 (19)	0
Hand-foot syndrome	3 (14)	1 (5)
Diarrhea	4 (19)	0
Hypothyroidism	3 (14)	0

## Discussion

Treating recurrent cervical cancer remains frustrating, both for patients and clinicians, particularly once standard options have been exhausted. In this retrospective analysis, the combination of fixed low-dose nivolumab with metronomic chemotherapy demonstrated encouraging clinical activity with a favorable toxicity profile.

Single-agent immune checkpoint inhibitors have previously shown response rates ranging from 12% to 26% in recurrent cervical cancer [3,4]. The CheckMate-358 study has shown an overall response rate of 26% with a single agent nivolumab, and the KEYNOTE-826 study has shown an overall response rate of 12% with pembrolizumab in PD-L1-positive patients [3,4].

Median overall survival of approximately 15 months is notable in a population that had already received definitive radiotherapy and platinum-based chemotherapy. While cross-trial comparisons should be made cautiously, these outcomes approach those reported in large randomized trials combining chemotherapy with full-dose immunotherapy such as CheckMate-358 and KEYNOTE-826, but at a fraction of the cost [9].

A study from India on the use of low-dose immunotherapy in recurrent/metastatic carcinoma of the cervix showed an overall response rate of 50%, of which half had a complete response, and the other half had stable disease [10]. They have used platinum agent or lenvatinib or bevacizumab as backbone combined with low-dose nivolumab, and this could be the reason for a slightly better overall response rate.

The observed benefit may reflect the immunomodulatory effects of metronomic chemotherapy. Low-dose methotrexate reduces regulatory T-cell activity, while metronomic capecitabine targets myeloid-derived suppressor cells, both of which may enhance PD-1 blockade [7,8].

In comparison, the overall response rate of 38% observed in our cohort appears higher than historical benchmarks, despite the use of substantially lower immunotherapy doses combined with chemotherapy.

It is important not to overinterpret these findings. The retrospective nature of the study, small sample size, and absence of biomarker stratification limit definitive conclusions. Nevertheless, the consistency of benefit across subgroups and the practical feasibility of the regimen support further investigation.

## Conclusions

Fixed low-dose nivolumab combined with metronomic methotrexate or capecitabine appears to be a reasonable and pragmatic option in carefully selected patients with recurrent cervical cancer, particularly where access to standard immunotherapy is limited. Further prospective studies are warranted to establish a firm benefit of this regimen.
